# Nursing professionals’ leading role in expanding the treatment for latent *Mycobacterium tuberculosis* infection in Brazil

**DOI:** 10.1590/1518-8345.0000.4083

**Published:** 2023-12-04

**Authors:** Fernanda Dockhorn Costa Johansen, Maria do Socorro Nantua Evangelista, Ethel Leonor Noia Maciel

**Affiliations:** 1 Coordenação-Geral de Vigilância da Tuberculose, Micoses Endêmicas e Micobactérias não Tuberculosas, Ministério da Saúde, Brasília, DF, Brasil.; 2 Unidade de Brasília, Faculdade de Ciências da Saúde, Departamento de Enfermagem, Brasília, DF, Brasil.; 3 Secretaria de Vigilância em Saúde e Ambiente, Ministério da Saúde, Brasília, DF, Brasil.

**Figure:**
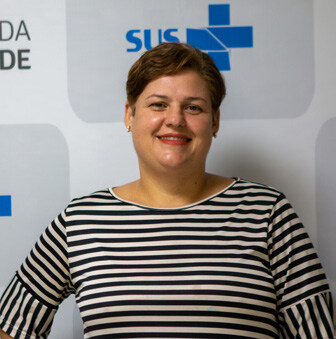


**Figure:**
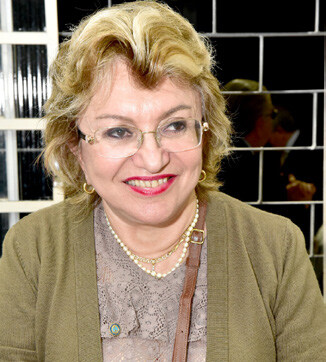


**Figure:**
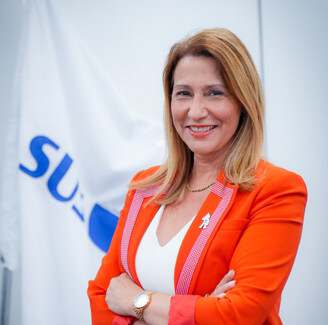


Brazil has made a high-level commitment to eradicate tuberculosis (TB) as a public health problem in the country by 2030 and, to achieve this goal, it becomes necessary to significantly expand investigation and treatment of latent *Mycobacterium tuberculosis* infection (LTBI), also known as Tuberculosis Preventive Treatment (TPT). 

Since the 1990s, the country has recommended investigating LTBI and TPT for contact children and people living with HIV; however, it was only in 2011 that this strategy was strengthened. Subsequently, following implementation of the Latent *Mycobacterium tuberculosis* Infection (LTBI) protocol in 2018, the Ministry of Health ( *Ministério da Saúde*, MS) considered the strategy as a priority but, despite all efforts, there was still low supply of LTBI treatment by services in Brazil ^(^ 1 ^)^ . 

Considering the *End TB* strategy, the World Health Organization (WHO) establishes that the LTBI treatment is fundamental for eradicating TB in the world ^(^ 2 ^)^ , being important to expand LTBI actions to reduce morbidity and mortality due to tuberculosis and the consequent future incidence of the disease by reducing the number of cases, particularly among contacts, immunocompromised people and the population living with HIV/AIDS. Hence the importance of increasing the LTBI strategy in Brazil and, thus, eradicating TB as a public health problem by 2035 ^(^ 3 ^)^ . 

To meet and achieve the operational goals (national and international) proposed in the National TB-Free Plan ^(^ 3 ^)^ , the importance of Brazilian Nursing in recent decades is highlighted, contributing and expanding its clinical practice (whether in the management of TB and/or LTBI cases), based on diverse evidence (in the use of investigation and research), in care management and planning ^(^ 4 ^)^ targeted at society’s individual and collective needs. In this sense, the care dimension in nurses’ work process in Primary Health Care for TB has gained strength and, when its practice is expanded, it is necessary to strengthen the “technical-scientific and political knowledge that does not materialize in isolation but requires interfaces with teamwork and interprofessional practice” ^(^ 4 ^)^ . Additionally, international experiences reinforce success of the treatment and clinical management of LTBI carried out by nurses ^(^ 2 ^)^ . 

In the meantime, aiming to change the scenario marked by low accessibility of people identified and treated for LTBI in the country, through the technical area of the General Coordination Office for the Surveillance of Tuberculosis, Endemic Mycoses and Non-Tuberculous Mycobacteria, belonging to the Health and Environmental Surveillance Department, the Ministry of Health held workshops with all Federated Units and identified the existence of multiple factors for the reduced indication of LTBI treatment and, among them, centralization of the prevention prescription in a single professional category, making it impossible to achieve the goals of expanding the LTBI indication, diagnosis and treatment strategy by the TB services. After this analysis, a new strategy was created with a view to optimizing the most effective TPT implementation in the services, with new duties for professional nurses.

It is worth noting the importance of Nursing professionals in the entire structure of the Unified Health System ( *Sistema Único de Saúde*, SUS), and also the historical relevance of the category’s leading role in the programmatic actions for TB control. This expertise supports the expansion of their professional performance, including requests for Interferon-Gamma Release Assays (IGRAs) in LTBI diagnosis and in performing TPT. 

Subsequently, a working group was assembled with several nurses, TB specialists, the TB Network and the Federal Nursing Council ( *Conselho Federal de Enfermagem*, COFEN), which jointly discussed the importance and possibility of these professionals diagnosing and treating LTBI, based on the COFEN’s consent. After an extensive debate, the COFEN issued a favorable opinion, strengthening the implementation of this strategy in the country ^(^ 5 ^)^ . 

Additionally, in the process of implementing the LTBI protocol carried out by nurses, the TB Network participates as a partner in expanding its contribution to nurses’ technical training in LTBI, in addition to being able to assist in increasing this strategy in the country, disseminating knowledge about TB prevention in order to organize the services, and the COFEN in regulating and disseminating the indication for latent infection treatment by nurses, at all care levels Finally, later this year, the Ministry of Health will formalize a clinical management protocol, with participation and actions targeted at nurses.

There is a major challenge ahead, following approval of COFEN’s Opinion No. 040/2023 ^(^ 5 ^)^ , with the need to establish new standards, protocols and training options for Nursing professionals, including care centered on people with TB in their work process ^(^ 4 ^)^ ; by strengthening prevention, diagnosis and follow-up of LTBI cases, as well as optimizing actions that ease adherence to the preventive treatment, in this effort by nurses to eradicate TB as a public health problem in Brazil. 
